# Progress in Medicine

**Published:** 1900-11-24

**Authors:** 


### Progress in Medicine.

**Respiratory System—Diseases of the Pleura.**—Simple pleural effusions may be due either to the action of bacteria or their toxins. If naturally absorbed, the visceral and parietal layers of the pleura become coated with fibrin, adhere together, and permanently obliterate the pleural sac. Our methods of hastening this absorption are mostly ineffectual, and recourse is made to aspiration. This rapid withdrawal of fluid is unsatisfactory, inasmuch as no layer of fibrin is deposited and the pleural sac may never be obliterated. Lewis[1] proposes injecting some easily diffusible antiseptic irritant into the sac without drawing off the fluid, so hastening absorption and ensuring a deposit of fibrin on the pleural surfaces. Failing to find a suitable medium, he determined to draw off 100 c.cs. of serum, mix the chemical irritant with this, and then return the whole to the chest cavity. Methylene blue was found the most efficacious drug, and 15 grains of this were added to every 100 c.c. of serum. In the twenty cases experimented upon the result was uniformly good, both as regards absorption of the fluid and obliteration of the sac. No pain or rise of temperature was experienced. As the great majority of simple cases of pleural effusion are due to the tubercle bacillus, it seems likely that the bactericidal properties of methylene blue play a great part in the result obtained.

Hæmorrhagic effusions into the pleura are generally due to cancer or tubercle. Fernet,[2] however, reports a case in which both of these could be excluded. The patient, an alcoholic man of fifty years, had suffered from dyspnœa and abdominal ascites for some years. He presented all the signs of extensive effusion into the left chest, and was aspirated on three occasions and relieved, in all, of $4\frac{1}{4}$ litres of blood-stained fluid. He finally died of uræmia, and the post-mortem examination showed renal and pulmonary cirrhosis, hæmorrhagic ascites, and double pleurisy. All the lesions were attributed to alcoholic irritation. The condition had previously been noted associated with cirrhosis of the liver. A similar case is reported by Cheesman and Ely[3] in a woman suffering from uterine fibroids and menorrhagia. She had a deeply blood-stained effusion into first the right and then the left pleural cavity. These were tapped and did not recur, but subsequently she suffered from hæmorrhagic ascites, which necessitated tapping on over forty occasions. The effusion ceased with the menopause.

Wilson[4] gives the following figures in connection with the bacteriology of empyema. Of all cases the infection is due to pneumococcus in 29 per cent., streptococcus 46 per cent., saprogenic forms 13 per cent., and tubercle 11 per cent. Pneumococcus infection is commonest in children, streptococcus in adults. Only 10 per cent. of cases were tubercular. Staphylococci are frequent in mixed infections, and cases of typhoid origin are recorded. The pus in pneumococcus cases is thin, and contains much fibrin either in the form of flocculi or pseudo-membrane; tubercular pus has a sanious, watery appearance. Cases may be classified as acute and subacute. The former are ushered in by a rigor, cough, dyspnœa, pain in the side, high temperature, and marked constitutional symptoms. In old standing cases with an extreme septic condition of the patient aspiration only is justifiable, the operation of resection being postponed until the general condition improves. Without resection of rib, drainage is seldom satisfactory; adhesions cannot be broken down, and digital exploration is difficult. The most advantageous spot is over the seventh or eighth rib in the post-axillary line. If necessary, the cavity may be flushed at the end of a week with sterile water or with 1–4000 permanganate of potash. Prognosis is favourable. König reports 12 deaths in 76 cases operated on. In these cases of empyema in which the lung does not expand two operations are possible: Estlander's, in which subperiosteal removal of a number of ribs is practised; and Schede's, in which a number of ribs are removed together with the periosteum. Two cases treated by the latter operation are recorded by Rutherford,[5] and it is noteworthy that in both instances there was atrophy of the abdominal muscles due to injury to the intercostal nerves. One case only showed slight lateral curvature of the spine.

Achambault[6] reports a case of empyema in a boy six years of age. In addition to the usual signs, there existed a pulsating, soft, and fluctuating tumour over the seventh, eighth, and ninth ribs, about midway between the axillary and scapular lines on the left side. The empyema was opened and drained through the pulsating area, and the child made a good recovery, the lung expanding perfectly. This result is exceptional, as in these cases of pulsating empyema the lung is generally permanently contracted, the heart displaced, and the prognosis very grave. The presence of a large effusion, the absence of murmur, thrill, or pressure symptoms, and the situation of the swelling differentiate the condition from aneurysm.

**Pneumothorax.**—Morse[7] gives the following conclusions from an analysis of fifty-one cases of pneumothorax. Seventy per cent. of all cases are tubercular; other exciting causes are trauma, pneumonia, abscess of lung, gangrene of lung. If secondary to trauma or abscess of lung the prognosis is fair; serous cases do better than purulent, right-sided better than left-sided. About 15 per cent. of all cases recover. Tubercular pneumo-thorax is most common in men than in women, and is most frequent in the third decade. It may be one of the first symptoms of tuberculosis noted. In about one-half of the cases the onset is acute, with sudden pain and dyspnœa. Displacement of the heart always occurs. Some cases have no fluid throughout.

Finley[8] records a case of sub-diaphragmatic abscess rupturing into the pleura, with subsequent production of gas by Bacillus coli and B. proteus. No source for the abscess was found. Other observers have recorded cases of gas formation in the pleural sac due to various organisms such as B. capsulatus aërogenes, B. coli, and Staphylococcus pyogenes. The presence of gases such as hydrogen not found in the atmosphere is conclusive proof of this condition being induced by gas-producing bacteria.

**Emphysema.**—Banti[9] says that the initial cause of emphysema is the deficient nutrition of the pulmonary walls brought about either by atheroma of the pulmonary artery or by acute diseases, such as pneumonia, affecting the lung. It is never a primary disease caused by unusual strain, but heredity apparently plays a part. Neither the inspiratory or the expiratory theory of production is sufficient to explain all cases, and he attributes the disease to the action of both these causes. Gee,[10] on the other hand, believes that pulmonary emphysema is chiefly due to forced inspirations, which first distend the chest walls. The sequence of events in his opinion is as follows:—First, an obstruction of the air passages, due to some form of bronchitis; secondly, a consequent feeling of dyspnœa, leading to violent inspiratory efforts; thirdly, a distension of the chest following these efforts, and leading to distension of the air vesicles. Once the air vesicles are over-distended the condition tends to become worse, for, owing to insufficient expiration, the blood is insufficiently aërated, and the dyspnœa continues. The over-distension of the air vesicles leads eventually to the degeneration and atrophy of lung tissue.

**Pulmonary œdema.**—A discussion upon this subject was opened at the thirteenth International Congress of Medicine by Professor Basch.[11] He showed that, given an elevation of pressure in the left auricle, and afflux of blood coming from the right heart, there must follow enlargement of the pulmonary alveoli and increased resistance in their walls, and to these is added transudation into the alveoli. These factors create a mechanical obstacle to respiration, leading to dyspnœa, which goes on to death from asphyxia. Cardiac dyspnœa, cardiac asthma, and pulmonary œdema have their starting point in weakness of the left auricle. Maxius rejected the above mechanical theory, and regards the exudation either as (1) an active secretion of the alveolar endothelium (inflammatory œdema). (2) Œdema of stasis, as in disease of heart, vessels, or kidneys. (3) Toxic œdema, as experimentally produced by muscarine or by iodine. The clinical features of this condition were dealt with by Teissier as being tickling in the throat and oppression in chest, dyspnœa, spasmodic cough, foamy and pinkish expectoration. Ausculation reveals very fine râles all over the chest. Pulmonary œdema is more common in persons recently recovering from infectious disease, in alcoholics, and in persons suffering from cardiac and nephritic disorders. The exciting cause is chill, fatigue, or emotion. Prognosis depends largely upon the kidney condition. Bleeding generally gives relief, and should be combined with counter-irritation over the heart and nerve plexuses. Teissier administered carbonic acid per rectum, and this, together with wet-cupping and derivatives, gave good results.

[1] Med. Rec., Dec. 30, 1899. [2] Lancet, July 28, 1900. [3] Amer. Jour. Med. Sci., cxviii., Aug. 1899. [4] Post. Grad., Feb. 1900. [5] Glas. Med. Jour., Apr. 1900. [6] Alb. Med. An., Feb. 1900. [7] Amer. Jour. Med. Sci., May, 1900. [8] Mont. Med. Jour., Oct. 1899. [9] Clin. Mod. Ann., n. 33. [10] Phil. Med. Jour., iii., No. 13. [11] Brit. Med. Jour. Ep. 98 et seq., Aug. 11, 1900.

